# The NRF2 transcriptional target *NQO1* has low mRNA levels in *TP53*-mutated endometrial carcinomas

**DOI:** 10.1371/journal.pone.0214416

**Published:** 2019-03-25

**Authors:** Guillaume Beinse, Pierre-Alexandre Just, Bastien Rance, Brigitte Izac, Franck Letourneur, Nathaniel Edward Bennett Saidu, Sandrine Chouzenoux, Carole Nicco, François Goldwasser, Eric Pasmant, Frederic Batteux, Bruno Borghese, Jérôme Alexandre, Karen Leroy

**Affiliations:** 1 INSERM U1016, Cochin Institute, CARPEM, Paris, France; 2 Paris Descartes University, Sorbonne Paris Cité, Paris, France; 3 Department of Pathology, Cochin Hospital, Assistance Publique–Hopitaux de Paris, Paris, France; 4 Department of Medical Informatics, HEGP, Assistance Publique–Hopitaux de Paris, Paris, France; 5 INSERM U1016, Cochin Institute, GENOMIC platform, Paris, France; 6 Department of Medical Oncology, Cochin Hospital, Assistance Publique–Hopitaux de Paris, Paris, France; 7 Department of Genetics and Molecular Biology, Cochin Hospital, Assistance Publique–Hopitaux de Paris, Paris, France; 8 Department of Immunology, Cochin Hospital, Assistance Publique–Hopitaux de Paris, Paris, France; 9 Department of Gynecologic Surgery, Cochin Hospital, Assistance Publique–Hopitaux de Paris, Paris, France; Sapporo Ika Daigaku, JAPAN

## Abstract

**Background:**

NRF2 is a major transcription factor regulating the expression of antioxidative/detoxifying enzymes, involved in oncogenic processes and drug resistance. We aimed to identify molecular alterations associated with NRF2 activation in endometrial carcinoma (EC).

**Methods:**

Ninety patients treated (2012–2017) for localized/locally advanced EC were included in this study. Formalin-fixed paraffin-embedded tissue samples were processed for immunohistochemical (NRF2 and Mismatch Repair proteins) analyses. Next generation sequencing (NGS) of a panel of genes including *POLE*, *TP53*, *NFE2L2*, *KEAP1* and *CUL3* was performed using Ampliseq panels on Ion Torrent PGM (ThermoFisher). NRF2 activity was assessed by *NQO1*, *GCLC*, and *AKR1C3* mRNA expressions, using TaqMan assays and quantitative RT-PCR.

**Results:**

Tumors were classified as *POLE* exonuclease domain mutated (*N* = 3, 3%), MMR-deficient (MSI-like) (*N* = 28, 31%), *TP53* mutated (Copy-number high-like) (*N* = 22, 24%), and other tumors (Copy-number low-like) (*N =* 32, 36%). NRF2 nuclear immunostaining did not correlate with NRF2 target genes expression. The 3 tumors with highest NRF2 target genes expression harbored oncogenic *KEAP1* or *NFE2L2* mutations. Low *NQO1* mRNA and protein levels were observed in the *TP53* mutated subgroup compared to others tumors (p < .05) and *in silico* analyses of The Cancer Genome Atlas data further indicated that NQO1 mRNA levels were lower in serous compared to endometrioid copy-number high EC.

**Conclusion:**

In contrast with previous reports based on immunohistochemistry, our study indicates that NRF2 activation is a rare event in EC, associated with *NFE2L2* or *KEAP1* mutations. The subset of aggressive EC with low *NQO1* mRNA level might represent a specific subgroup, which could be sensitive to combination therapies targeting oxidative stress.

## Introduction

Endometrial carcinoma is the most frequent gynecological cancer in woman. Two main histological types have been described, type 1 endometrioid carcinoma and type 2 including non-endometrioid subtypes (high grade serous, clear cell carcinoma, carcinosarcoma) with poorer prognosis [1]. This classification was refined in 2013 by an integrated genomic characterization [2], which allowed to identify four major molecular groups: 1/ an ultra-mutated group, with DNA-Polymerase ε (*POLE*) catalytic subunit A mutations; 2/ a hypermutated group, characterized by a somatic microsatellite instability (MSI), largely due to methylations in *MLH1* promoter; 3/ a group characterized by low copy-number alterations (Copy-number low group (CNL)); and 4/ a group characterized by high copy number alterations (Copy-number high group (CNH)) and *TP53* mutations, that includes most serous carcinoma and some high grade endometrioid histologic subtypes, and that carries the worst prognosis. Despite these new insights, therapeutic breakthroughs are still awaited.

Most tumor cells are characterized by their increased production of reactive oxygen species (ROS) within the mitochondrial respiratory chain in relation to high metabolic activity [3,4]. To regulate ROS homeostasis, tumor cells need to activate anti-oxidative stress (AOS) response elements, mainly through NRF2 activation, a transcription factor encoded by the gene *NFE2L2*. At basal levels, NRF2 is bound to its repressor, Kelch-like ECH-associated protein 1 (KEAP1), which drives its ubiquitination by CUL3 and subsequent degradation in the proteasome. Increase in cellular ROS levels induces the disruption of the NRF2/KEAP1 couple thereby allowing NRF2 translocation into the nucleus and its binding to AOS response element (ARE) promoters [5–7]. NRF2 activates the transcription of thousands of genes belonging to multiple pathways [6,8] involved in cell detoxification and in a cell metabolism reprogramming in which glucose is redirected in the pentose phosphate pathway.

Deregulated NRF2 activation in solid tumors has been largely reported and could drive cancer progression, metastasis, and resistance to therapy [9]. The disruption of NRF2 binding to KEAP1 can be due to mutations in the binding domains of NRF2 or KEAP1 [10–12]. NRF2 activation may also be induced by the loss of its negative regulation by the PTEN/GSK3β axis [13], which regulates beta-TRCP CUL1 degradation pathway [3,14]. Moreover, *NFE2L2* gene expression may be induced by the oncogenic activation of proliferative signaling pathways (such as *KRAS*, *BRAF*, *CMYC* mutations or overexpression) [15].

In clinical studies, NRF2 activation has been largely assessed by its nuclear translocation, using immunohistochemistry (IHC). In endometrial carcinoma, conflicting results were reported. Thus, NRF2 activation has been described as more frequent in serous carcinoma in one study [16], but was observed in a large subset of endometrioid carcinoma with PTEN loss in another one [13]. The main weakness of these studies was the restriction of the “*NRF2 activation*” definition to immunohistochemistry assays [17].

Our study aimed to identify molecular alterations associated with NRF2 transcriptional activation, and to explore its impact on prognosis, in a population of patients treated for localized or locally advanced endometrial carcinoma. Herein, we provide new insights allowing a better understanding about the role of NRF2 in endometrial carcinoma, and more particularly in aggressive *TP53/CNH* tumors, which contrasts with previous findings.

## Material and methods

### Endometrial carcinomas tissue selection and DNA extraction

The Cochin Hospital Endometrial Cancer cohort included all consecutive patients who underwent tumor biopsy or tumor resection in the Gynecological Department between 2012 and 2017 for uterine carcinoma, and for whom formalin-fixed paraffin embedded (FFPE) tissue samples were available for further analysis. Genomic DNA extraction was performed on the sample area with the highest cellularity, using the Maxwell 16 FFPE Plus LEV DNA Purification Kit (Promega, Charbonnières-les-Bains, France), according to the manufacturer’s instructions. Clinical and molecular analyses of the whole cohort are reported elsewhere (Beinse et al. *under submission*). For the present study, all patients with samples available for RNA extraction were considered. All patients gave written informed consent for somatic genetic analyses and data collection. This study was approved by the Paris ethic committee (CPPIDF1-2015—DAP22).

### Targeted sequencing using AmpliSeq and Ion Torrent technologies

Targeted amplification and library preparations were performed with Ion AmpliSeq protocols. Libraries were clonally amplified on sequencing beads in emulsion PCR with Ion OneTouch. Sequencing was performed using Ion Torrent technology with Ion Torrent Personal Genome Machine (PGM) System (Life Technology, ThermoFisher Scientific, Courtaboeuf, France).

The first gene panel included the following genes: *TP53*, *KRAS*, *PTEN*, *PIK3CA*, *PIK3R1*, *POLE*, *ERBB2*, *CTNNB1*, *RPL22*, *PPP2R1A*, *ARID1A*, *ARID5B*, *CTCF*, *FBXW7*, *FGFR2*. This panel was designed using AmpliSeq Designer (version 4.47) on Human genome *hg19*. Overall, 440 amplicons were designed in 2 pools (**S1 Method**). For the present study, we report results for genes identifying endometrial carcinoma molecular subgroups (*POLE* exonuclease domain and *TP53* mutations), and mutations in *KRAS* coding sequence or belonging to the PI3K pathway (*PTEN*, *PIK3CA*, *PIK3R1*). The second panel targeted the complete coding sequences of *NFE2L2*, *KEAP1*, *and CUL3* (**S2 Method**). Mean depth sequencing was >100X for all amplicons of *NFE2L2* and *KEAP1*, and for 37/42 amplicons of *CUL3*.

### Bio-informatics analysis of sequencing data

Torrent Suite Software (v. 5.6) was used for sequencing data processing. TMAP (Torrent Mapping Alignment Program) software was used to perform reads processing and mapping on loaded genome (Hg19), using default parameters. Samples with mean read length <90 bp, mean depth <150X, uniformity of coverage <70%, were removed in subsequent analysis (see **S1 Fig** for flow-chart). Variant Caller Plugin was used for variant calling. Variant Caller’s parameters were chosen to allow variant detection with minimal stringency, in order to get a high sensitivity, and to preclude false-negative (Parameters provided in **S3 Method**). COSMIC database reference transcripts were used for variant call. Variants were filtered to include in the final manual reviewing all significant (p<0.0001, based on Phred quality score logarithmic transformation), somatic non-synonymous variants (Global minor allele frequency <0.1% in 1000 Genomes Project database), with a coverage ≥ 50X. All selected variants were one by one reviewed manually (G.B. & K.L.) using the Integrative Genomics Viewer (IGV) tool [18,19] and automated annotations (dbSNP, 1000 Genome Project, UCSC common SNPs, COSMIC, Gene Ontology). Variants were assumed to be clonal or sub-clonal according to the analysis of the allele ratio distribution of all variants belonging to the same sample. Variants with lowest allele ratio reaching maximal Euclidean distance compared to all others were considered as sub-clonal.

Final annotations of KEAP1 and NFE2L2 mutations were performed manually on the basis of relevant literature, and using Cancer Genome Interpreter [20] (https://www.cancergenomeinterpreter.org) and OncoKB [21] (http://oncokb.org).

### Immunohistochemistry

#### Hormone receptors, TP53, and mismatch repair status

The following immunohistochemistry assays were performed in routine practice: hormone receptors (estrogen (ER) and progesterone (PR)), TP53, and MSH6 and PMS2 to estimate the mismatch repair status. Immunohistochemistry was performed on a Leica Bond-III Autostainer using the Bond Polymer Refine Detection Kit (Leica Biosystem), according to the manufacturer’s instruction. The following primary antibodies were applied on 5μm FFPE tissue slides after appropriate heat-induced epitope retrieval (ER1 or ER2, Leica Biosystems): TP53 (DO-7; DAKO; 1:800 dilution), PMS2 (A16-4; Pharmingen; 1:300 dilution), MSH6 (44; LSBio; 1:50 dilution), ER (1D5; DAKO; 1:50 dilution), and PR (PgR636; DAKO; 1:300 dilution). Staining was categorized using a standard pathological system, with the staining intensity (+ to +++) and the percentage of tumor stained cells (0 to 100%). Hormone receptors staining were considered positive if >+ and >10%, TP53 staining was considered abnormal if >+ and >10%, or in case of staining loss with positive endogenous control [22]. Mismatch repair deficiency was defined by the loss of staining of either PMS2 or MSH6 [23].

#### NRF2 and NQO1 staining

Immunohistochemistry was performed on a Leica Bond-III Autostainer, using the Bond Polymer Refine Detection System Kit (Leica Biosystem), according to the manufacturer’s instructions. Epitope retrieval was performed in ER2 buffer (EDTA based buffer and surfactant, pH = 9) for 30 min at 95°C for NRF2 staining and in ER1 buffer (EDTA based buffer and surfactant, pH = 6) for 20 min for NQO1 staining. Primary NRF2 antibody (Santa-Cruz Biotechnology A-10, sc-365949) was applied at 1:100 dilution for 1 h at room temperature. Primary NQO1 antibody (SIGMA-ALDRICH, HPA007308) was applied at 1:150 dilution for 20 min at room temperature. Slides were counterstained in hematoxylin and mounted in Pertex mounting medium (CellPath). Staining was categorized using a standard pathological system, with the staining intensity (+ to +++) and the percentage of tumor stained cells (0 to 100%). Samples were considered NRF2-high if nuclear staining intensity was +++ in more than 50% of tumor cells, and intermediate (+++/<50% and ++/>50%), low (++/<50% and +/>50%), or negative (+/<50% and 0).

### RNA extraction

RNA extraction was performed using the Maxwell 16 LEV RNA FFPE Kit according to the manufacturer’s instructions. Briefly, 10 μm sections from FFPE samples (maximum 1 mm^3^) were immersed in mineral oil and heated (5 minutes at 80°C) for deparaffinization. Samples were then lysed with proteinase K solution (15 minutes at 56°C, and 60 minutes at 80°C), treated with DNase I, and finally automatically processed on magnetic beads.

### Gene expression assay

Reverse transcription (RT) was performed using the High Capacity cDNA Reverse Transcription Kit (Applied Biosystem, ThermoFisher Scientific), with up to 1400 ng of RNA (or up to 14μL of RNA sample for samples with the least yields) in a 20μL RT reaction. Samples were incubated in 1X RT buffer, 4mM dNTP mix, with 1X random primers, and 2.5U Multiscribe Reverse Transcriptase, during 10 minutes at 25°C, 120 minutes at 37°C, and 5 minutes at 85°C. Complementary DNA (cDNA) obtained were diluted to 1:5 in RNase-free water (final volume: 100μL) before quantitative PCR (qPCR).

qPCR were performed using TaqMan Gene expression assays (ThermoFisher Scientific) in a 20μL reaction with LightCycler 480 Probes Master (Roche Diagnostics, Basel, Switzerland) on LightCycler 480 System Instrument (Roche Diagnostics), using 3 μL of cDNA, 1 μL of 20X TaqMan primer-probe mix, 10 μL of 2X LightCycler 480 Probes Master mix, and RNase-free water up to 20 μL. After incubation at 95°C for 10 minutes, the reactions underwent 45 cycles as follow: 10 sec at 95°C, 30 sec at 60°C, and 1 sec at 72°C,. Duplicate qPCR were performed on three core NRF2 target genes: *NQO1* (TaqMan probes ID Hs01045993_g1), *AKR1C3* (TaqMan probes ID Hs00366267_m1), and *GCLC* (TaqMan probes ID Hs00155249_m1), and two housekeeping genes: TBP (TaqMan probe ID Hs00427621_m1) and MRPL19 (TaqMan probes ID Hs00608519_m1). Nine samples showing more than 3 genes with Cq≥35 were excluded (**S1 Fig**). Quantifications results are presented as: DeltaCq = -[Cq_Gene_- mean(Cq_TBP_, Cq_MRPL19_)] so that positive values represent high expression and negative values represent low expression.

### Statistical analysis

Correlations between qualitative and continuous variables were analyzed using Student t-test, or, if inapplicable, non-parametric test (Wilcoxon test). Correlations between continuous variables were analyzed using logistic regression. Correlations between qualitative variables were assessed using Fisher’s exact test. Clustering analysis based on NRF2 target genes expressions used principal component analysis. Survival analyses were assessed by logistic regression using the Cox regression model. Primary event of interest was event free survival (EFS), defined as any event (progression, relapse, or death), censored by date of last news. Evaluation of the proportional hazard assumption was based on the Schoenfeld residuals method. Kaplan-Meier survival analysis used two-sided log-rank test. Statistical significance was defined by *p*<0.05. All analyses were performed using R software v3.3.3.

## Results

### Classification of endometrial carcinoma according to TCGA molecular subgroups

Among 159 consecutive endometrial carcinoma patients treated at Cochin Hospital between 2012 and 2017, 125 patients had tumor DNA available for molecular analyses and 90 could also be analyzed at the RNA level (**S1 Fig**). Tumor classification was based on *POLE* exonuclease domain mutation, mismatch repair protein loss of expression and *TP53* mutation, to retrieve TCGA molecular subgroups [2], as described in previous studies[24,25]. Population characteristics and tumor’s features are provided in **Table 1**. Overall, 3 out of 90 tumors (3.3%) harbored a mutation into the POLE exonuclease domain, defining *POLE* mutated tumors (*ultramutated*-*like*); 28 tumors (31.1%) had lost expression of PMS2 or MSH6, defining MMR deficient tumors (*MSI-like*); 22 (24.4%) tumors were mutated within the *TP53* coding sequence, defining a TP53 molecular group (*CNH*-*like*). Finally, 32 tumors (35.6%) had no aforementioned alteration, defining a copy-number low-like group (*CNL-like*). The distributions of histological types and molecular groups were consistent with endometrial carcinoma epidemiology, and previously published data [1,2].

**Table 1:** Clinical and pathological characteristics in the overall population and according to NRF2 nuclear staining.

	Overall population N = 90	NRF2 nuclear staining^a^ (N = 89)		*p*^*b*^
		High	Others	
**Age at surgery or diagnostic**, median [min –max] (years)	66.2 [44.0–94.3]	-	-	-
**Histological main type**, N (%)				0.80
**Type I carcinoma**	73 (81.1%)	17 (23.6%)	55 (76.4%)	
Grade I endometrioid carcinoma	42 (46.6%)			
Grade II endometrioid carcinoma	24 (26.7%)			
Grade III endometrioid carcinoma	7 (7.8%)			
**Type II carcinoma**	17 (18.9%)	5 (29.4%)	12 (70.6%)	
Serous carcinoma	10 (11.1%)			
Carcinosarcoma	5 (5.6%)			
Clear cell carcinoma	2 (2.2%)			
**Tumor FIGO stage**^c^, N (%)				0.85
**Localized**	61 (67.8%)	16 (26.7%)	44 (73.3%)	
IA	31 (34.4%)			
IB	29 (32.2%)			
II	1 (1.0%)			
**Locally advanced / metastatic**	27 (30.0%)	6 (22.2%)	21 (77.8%)	
IIIA	2 (2.2%)			
IIIB	1 (1.0%)			
IIIC1	5 (6.0%)			
IIIC2	12 (13.3%)			
IVB	7 (7.7%)			
Missing data	2			
**Lymphatic or vascular invasion**, N (%)				0.23
No	61 (67.8%)	18 (30.0%)	42 (70.0%)	
Yes	24 (28.2%)	3 (12.5%)	21 (87.5%)	
Missing data ^d^	5			
**Hormone receptors**, N (%)				
**Estrogen receptor**				0.79
< 10%/+	15 (16.7%)	3 (20.0%)	12 (80.0%)	
≥ 10%/+	75 (83.3%)	19 (25.7%)	55 (74.3%)	
Missing data	0			
**Progesteron receptor**				1.00
< 10%/+	33 (37.9%)	7 (21.2%)	26 (78.8%)	
≥ 10%/+	54 (62.1%)	12 (22.6%)	41 (77.4%)	
Missing data ^d^	3			
***TP53* immunostaining**, N (%)				0.39 ^e^
Normal (*Wild-type-like*)	61 (67.8%)	15 (24.6%)	46 (75.4%)	
Overexpression	24 (26.7%)	6 (26.1%)	17 (73.9%)	
Loss of expression	2 (2.2%)	0 (0.0%)	2 (100.0%)	-
Missing data ^d^	3	1	2	
**Molecular group** ^f^, N (%)				0.13 ^g^
POLE (*ultramutated-like*)	3 (3.3%)	0 (0.0%)	3 (100.0%)	-
MMR deficient (*MSI-like*)	28 (31.1%)	4 (14.3%)	24 (85.7%)	
TP53 (*CNH-like*)	22 (24.4%)	8 (36.4%)	13 (59.0%)	
MMR proficient (*CNL-like*)	32 (35.6%)	9 (28.1%)	23 (71.9%)	
Missing data ^d^	5 (4.4%)	1	4	
**Total**	90 (100%)	22 (24.7%)	67 (75.3%)	

^a^ data missing for one patient (see #); NRF2-high: staining intensity of +++ in more than 50% of tumor cells.

^b^ p-values estimated with Fisher exact test.

^c^ International Federation of Gynecology and Obstetrics (FIGO) 2010 staging.

^d^ data missing because of technically uninformative immunostaining test. </≥ 10%/+: hormone receptor staining intensity ≥ + in ≥ 10% of tumor cells.

^e^ Fishers test was performed without consideration of the group with loss of expression, because there was no subject in one group.

^f^ based on *POLE* and *TP53* targeted sequencing and mismatch repair (MMR) proteins immunostaining–MSI: microsatellite instability. CNH: copy-number high. CNL: copy-number low. Tumors with a mutation in the *POLE* exonuclease domain are classified as POLE tumors, tumors with a defect of expression of one MMR protein were classified as MMR deficient (microsatellite instable-like) tumors, tumors with *TP53* mutations are classified as TP53 molecular group (copy number high-like), and all others are classified as MMR proficient (copy number low-like) tumors.

^g^ Fisher test was performed using molecular subgroup as one categorical variable, without consideration of *POLE* because there was no subject in one group.

### NRF2 expression and transcriptional activity in endometrial carcinoma

**Fig 1:**
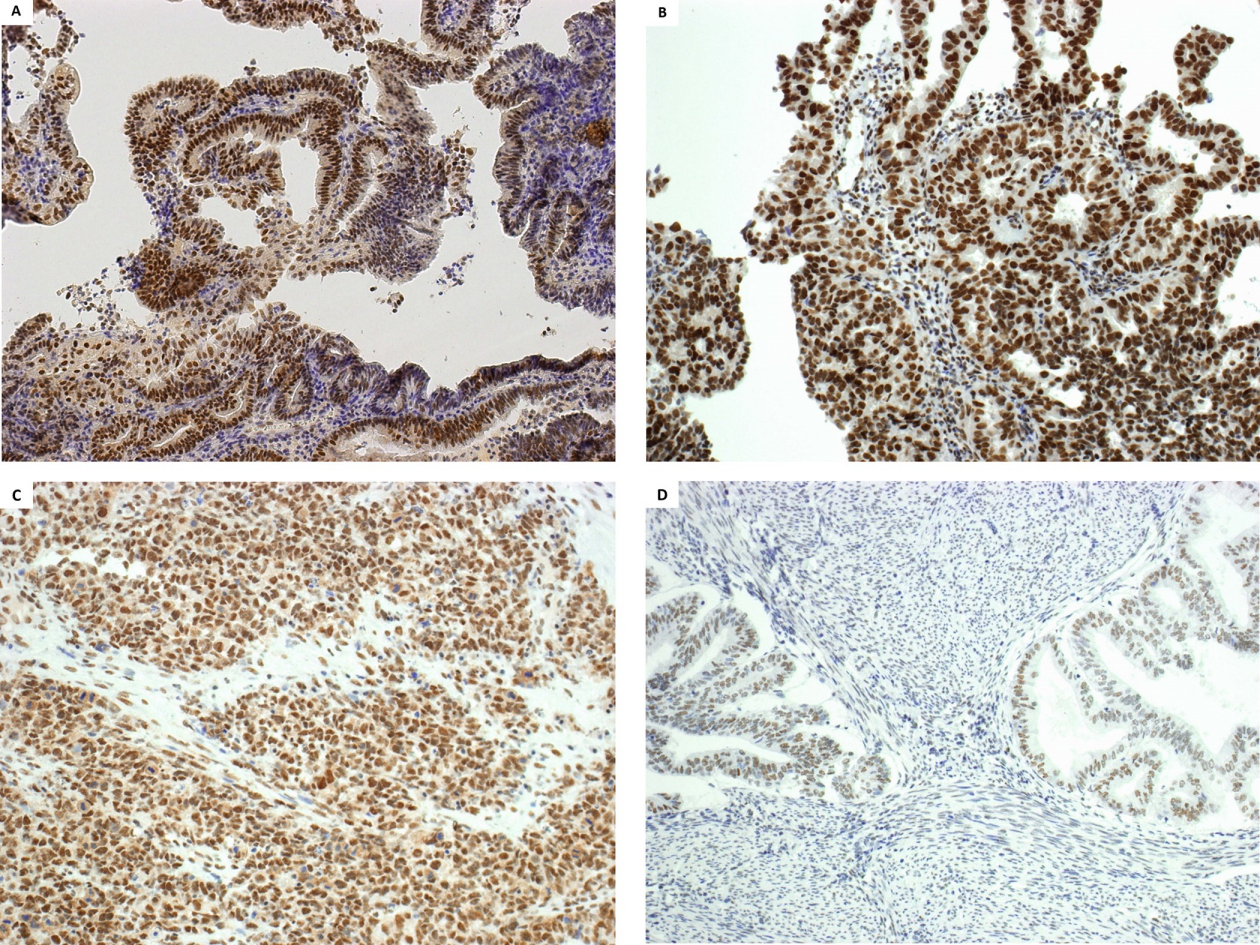
Representative NRF2 immunostaining in endometrial tumors. Immunohistochemical assay using NRF2 (clone A-10, SantaCruz) monoclonal antibody, revealed with Leica Bond Polymer Refine Detection System Kit. 10X objective. **A**: endometrioid grade I, *copy number low*-like tumor–stage IB. NRF2 *high* case. **B**: serous carcinoma *copy-number low*-like tumor, stage IIIC2 –NRF2 *high* case. **C**: carcinosarcoma, *copy number high*-like tumor–stage IB. NRF2 *intermediate* case. **D**. endometrioid grade I, *copy number low-*like tumor–stage IB. NRF2 *negative/low* case. Note that: **A**: sample with NRF2 activating mutations (*KEAP1* p.R336*).

We assessed NRF2 nuclear staining using immunohistochemistry on FFPE sections (**Fig 1**). Nuclear staining was not observed into the nuclei of normal epithelial and stromal cells. For some surgical specimens, we observed a variability of NRF2 staining in tumor cells within the same section, possibly due to formalin-fixation artifacts for large surgical specimens. Therefore, for these specimens, NRF2 staining was considered only for morphologically well-fixed regions (i.e. without morphological degradation). When scoring the staining by intensity (+ to +++) and extension (0 to 100% of tumor cells), we were able to identify a subset of tumors with high NRF2 nuclear staining (+++ in more than 50% of tumor cells) (*N =* 22, 24.7%) (**Table 1**). No tumor pathological nor clinical features was significantly associated with this high NRF2 nuclear staining (**Table 1**) although we observed a trend towards a higher proportion of NRF2-high tumors in the *TP53*/*CNH*-like tumors (36.4% versus 19.4% in non-*TP53*/*CNH*-like tumors, *p* = 0.052).

**Fig 2:**
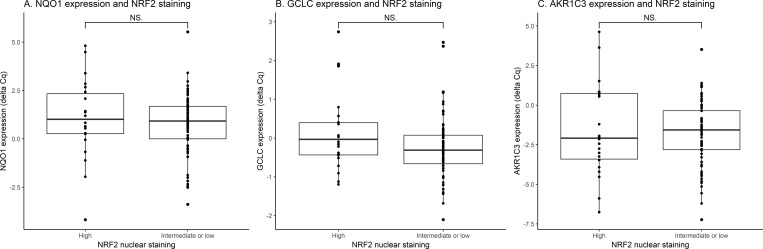
NRF2 target genes expression and NRF2 immunostaining in endometrial tumors. *NQO1*, *GCLC* and *AKRC3* expressions measured by quantitative RT-PCR are shown as interquartile boxplots in tumors with high or with intermediate/low NRF2 nuclear staining. NS: non-significant. *p*-values computed using Wilcoxon test.

As NRF2 is a transcription factor with well-known target genes [3,4], we analyzed the mRNA levels of three of its target genes (*NQO1*, *GCLC*, *AKR1C3*). *NQO1* was chosen because involved in phase II xenobiotic detoxification and in NADPH consumption. *GCLC* was chosen because being involved in glutathione production, considered as the most abundant anti-oxidant cellular cofactor. Finally, aldo-keto-reductase genes have been reported to be among the most inducible NRF2 targets in human systems [26]. Overall, *NQO1*, *AKR1C3* and *GCLC* mRNA levels of expression were correlated with each other (Pearson correlation coefficient for *NQO1/AKR1C3* = 0.41, *NQO1/GCLC* = 0.41 and *GCLC/AKR1C3* = 0.55) and highly variable within the whole cohort (**S2 Fig**). No statistical association was observed between NRF2 high nuclear staining and NRF2 target genes expression (**Fig 2**), indicating that nuclear staining is not representative of NRF2 activity and appears as a poor surrogate marker of NRF2 transcriptional activation in these tumors.

### *NFE2L2/KEAP1* mutations in endometrial carcinoma and NRF2 target gene expression

Molecular alterations within the NRF2 pathway were assessed using next-generation sequencing with a dedicated panel that included *NFE2L2*, *KEAP1*, and *CUL3* coding sequences. With a mean coverage of 880X per sample, 1924 variants were identified. After filtering for germline polymorphisms, non-coding and silent variants, and low-quality calls, 127 variants were selected for manual reviewing. After exclusion of sequencing artifacts, 16 variants (**Table 2**) were considered for manual annotation based on literature [10,12,27] and database annotation (Cancer Genome Interpreter [20] and OncoKb [21]). Eight variants were identified in *NFE2L2*, among which 3 (NRF2 p.W24G, p.E82D and p.G81V) were located at known hotspot positions known to disrupt KEAP1 binding. Among the five other variants, four were annotated as passenger mutations. The last one (NRF2 p.R499W) was predicted *in silico* as driver, nevertheless the absence of supporting experimental evidence and its association with deleterious *KEAP1* variants precluded a definitive assessment regarding its pathological function. Five variants were found in *KEAP1*, among which 2 were expected to affect NRF2 binding: p.D422N, which is a recognized oncogenic mutation and p.R336*, which is a non-sense truncating mutation. For the 3 other variants, there was no available functional data that support a deleterious function, although 2 of them (p.G350R and p.G509R) were located in the protein domains involved in NRF2 binding. Of note, 2 samples harbored several variants and some variants were suspected to be sub-clonal considering the distribution of variant allele frequency within the tumors (**Table 2**). Overall, 5 samples harbored known NRF2 pathway activating mutations. The 3 samples with *CUL3* mutations did not harbor high NRF2 target expression, or had concomitant NRF2-activating mutations.

**Table 2:** NRF2 pathway mutations and NRF2 target gene expression.

Patient	Histological type	Molecular subgroup	NQO1 deltaCq	GCLC deltaCq	AKR1C3 deltaCq	*GENE*, variant	Allele ratio	CGI	Onco Kb	Affects domains known to be involved in NRF2/KEAP1 binding^27^	Known association with NRF2 pathway activation	Comments and references
A	Mucinous	MSI	5,53	2,47	3,52	*NFE2L2*, p.W24G	34%	Tier 1	*aa 24 *: *LO*	Yes (DLG motif)	*Yes*	Located at *NFE2L2* Exon 2 hotspot (although different aminoacid change)^12^
B	Endometrioid (grade I)	CNL	4,81	2,74	4,63	*NFE2L2*, p.R499W	37%	Tier 1	-	No		
						*KEAP1*, p.R336*	39%	*Passenger*	*-*	Yes	*Yes*	*KEAP1* truncating mutation^10^
						*KEAP1*, p.G509R	37%	Tier 1	*-*	Yes		
C	Serous	TP53	4,48	1,91	3,64	*KEAP1*, p.D422N	75%	Tier 1	*O*	Yes	*Yes*	KEAP1 mutation in the NRF2/KEAP1 binding domain^12,27^
D	Endometrioid (grade I)	MSI	3,41	0,88	1,27	*KEAP1*, p.R169H	51%	Tier 1	-	No		
						*CUL3*, p.Q188H	10%	Passenger	-	No		
						*KEAP1*, p.G350R	50%	Passenger	*-*	Yes		
						*NFE2L2*, p.E82D	14%	Tier 1	*LO*	Yes (ETGE motif)	*Yes*	*NFE2L2* Exon 3 hotspot–suspected subclonal^12^
E	Endometrioid (grade II)	MSI	3,39	0,41	0,77	*NFE2L2*, p.S137L	13%	Passenger	-	No		
F	Endometrioid (grade I)	MSI	2,42	0,36	0,65	*NFE2L2*, p.G81V	16%	Tier 1	*LO*	Yes (ETGE motif)	*Yes*	*NFE2L2* Exon 3 hotspot–suspected subclonal^12^ †
G	Endometrioid (grade II)	POLE	2,27	0,94	0,31	*NFE2L2*, p.K599Q	12%	Passenger	-	No		
H	Endometrioid (grade II)	MSI	1,90	-1,40	-7,23	*CUL3*, p.D121E	42%	Tier 1	-	No		
I	Endometrioid (grade III)	POLE	1,71	0,23	-1,41	*NFE2L2*, p.S137L	8%	Passenger	-	No		
J	Endometrioid (grade II)	MSI	0,92	-0,29	-1,87	*CUL3*, p.R588Q	28%	Tier 1	-	No		
K	Endometrioid (grade III)	CNL	-0,74	-0,50	-2,78	*NFE2L2*, p.Q171R	25%	Passenger	-	No		

CGI: Cancer Genome Interpreter (https://www.cancergenomeinterpreter.org/home); Tier 1: predicted driver with high level of stringency based on OncodriveMUT interpretation on CGI. OncoKB: http://oncokb.org; LO: likely oncogenic, according to OncoKB annotation; O: oncogenic, according to OncoKB annotation. aa: aminoacid. Boxes colored in grey highlight values in upper quartile in the expression distribution of the considered gene. POLE: *ultramutated-like* tumors, with mutation within the *POLE* exonuclease domain; MSI: *MSI-like tumors*, with mismatch repair system protein loss of expression; CNL: *copy-number low-like* tumors; TP53: *copy-number high-like* tumors, without POLE mutation or mismatch repair system deficiency, while affected by *TP53* mutation. Refseq sequences used for variant call: *NFE2L2*: NM_006164.4; *KEAP1*: NM_203500.1; *CUL3*: NM_003590.4.

**Fig 3:**
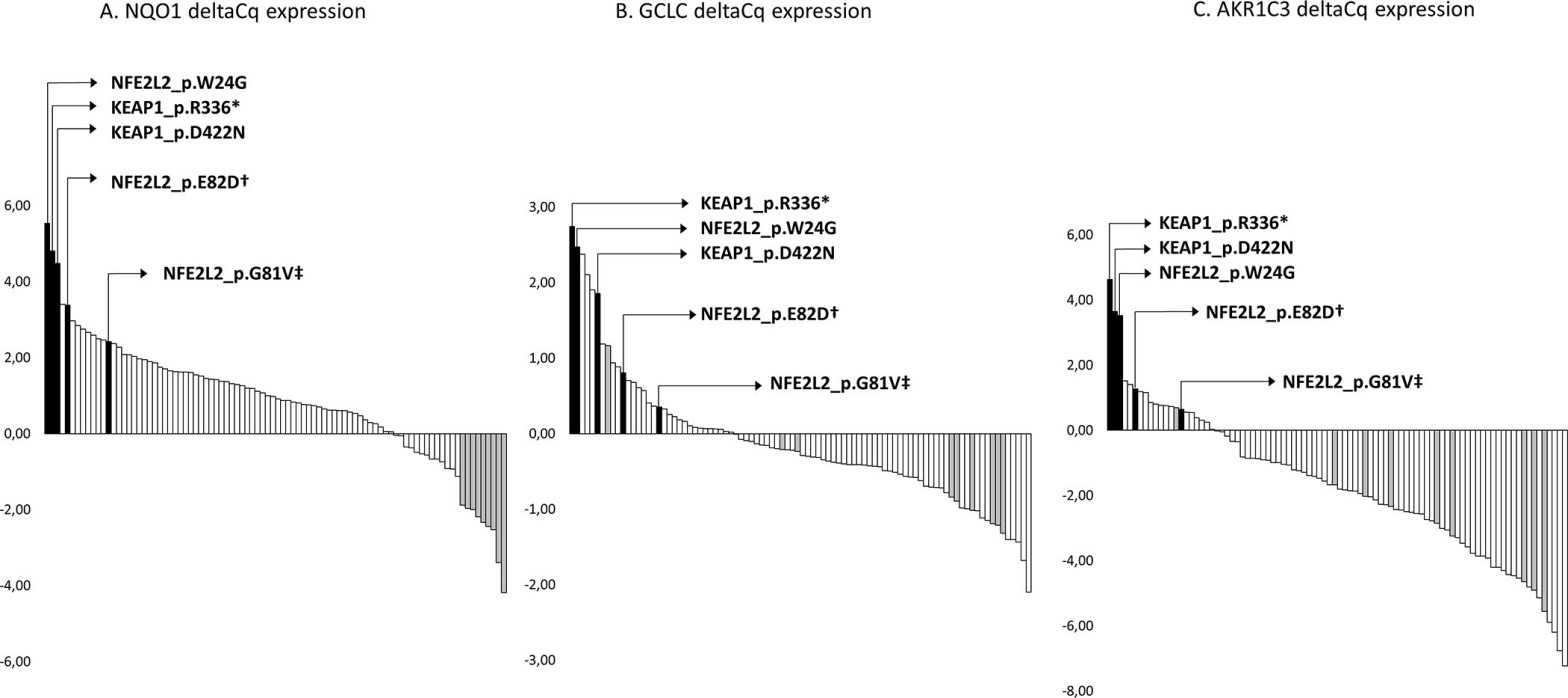
Distribution of NRF2 target genes expression and *NFE2L2/KEAP1* mutations in endometrial tumors. NQO1, GCLC and AKRC3 expressions measured by quantitative RT-PCR are shown as waterfall plots. *y-*axis display delta Cq expression. Black bars: samples affected by NRF2 pathway activating mutations. Grey bars: samples considered as outliers regarding NQO1 low expression, based on the observation of a gap into the NQO1 expression distribution which mirrors the gap observed for the samples with high NQO1 expression. Grey bars in GCLC and AKR1C3 distribution depict corresponding samples. † NFE2L2 p.E82D with allele ratio = 14%, and ‡ NFE2L2 p.G81V with allele ratio = 16%, suggesting of a subclonal NRF2 activation. See Table 2 for details on NRF2 pathway mutations and annotations.

The three samples with high mRNA levels for all three NRF2 target genes harbored mutations affecting the NRF2/KEAP1 binding (**Fig 3,** and **Table 2**). NRF2 nuclear staining of these three tumors was considered as high (*N =* 2) or intermediate (*N =* 1). Two other samples with likely oncogenic *NFE2L2* variants at low allele ratios had an intermediate/high expression of these three target genes (first quartile). Because of the limited number of samples, no formal relevant statistical test could be performed. However, using a principal component analysis based on these three NRF2 target genes expression to cluster samples, we found an overlap between samples belonging to the four molecular subgroups, contrasting with a distinct cluster including the three samples harboring clonal NRF2 activating mutations (*NRF2 group*) (**S3 Fig**). The same type of analysis was applied to the TCGA cohort data, highlighting the existence of a small group of tumors with pathogenic *NFE2L2/KEAP1* mutations and high NRF2 target genes expression (**S4 and S5 Figs**). These data indicate that strong NRF2 transcriptional activation in endometrial carcinoma is related to specific *NFE2L2/KEAP1* mutations and independent of the molecular endometrial carcinoma subgroups.

### Molecular and histo-pathological features associated with NQO1 mRNA level

**Fig 4:**
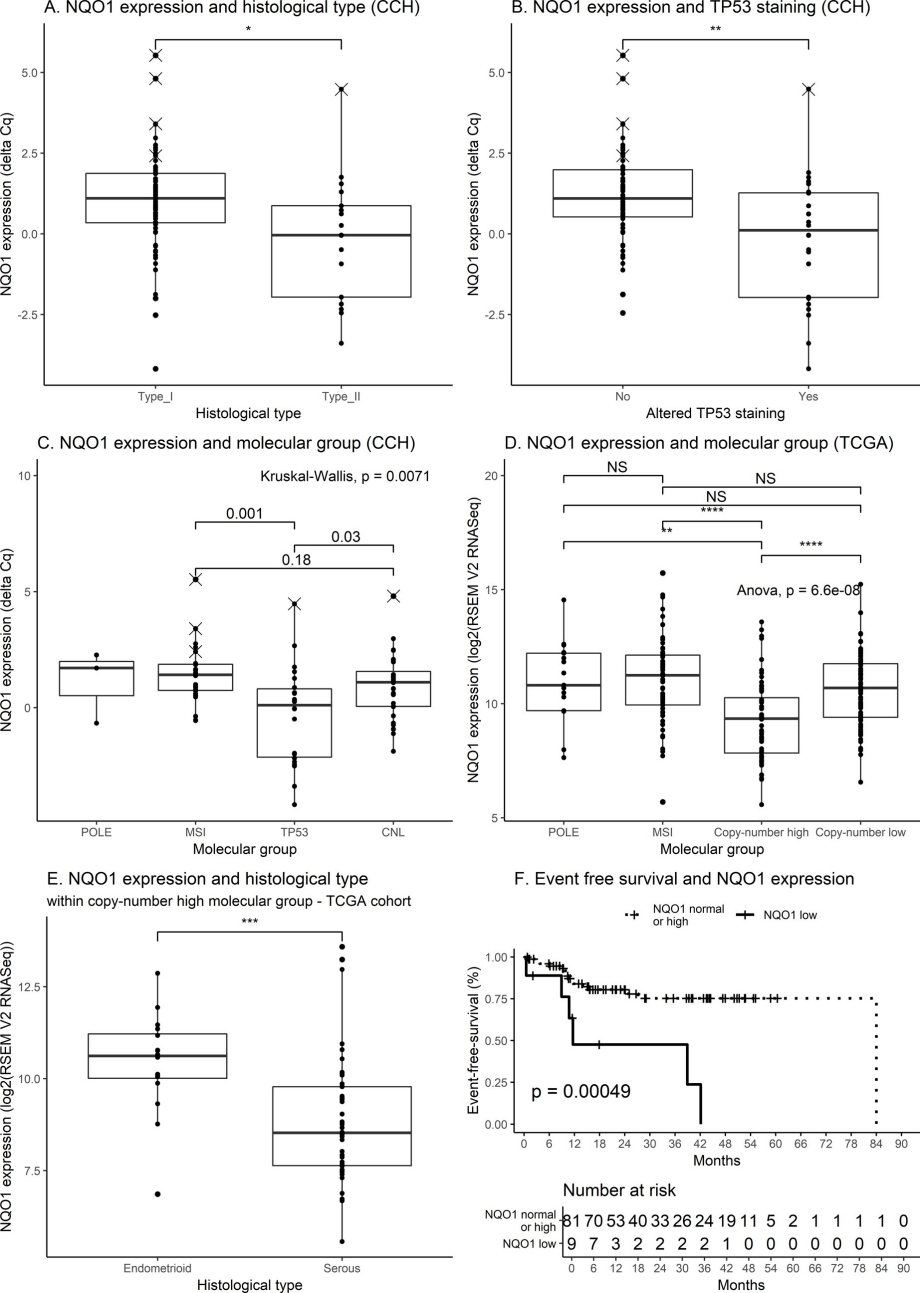
NQO1 expression in endometrial carcinoma. NQO1 expression was measured by quantitative RT-PCR in Cochin Hospital cohort (CCH) and RNAseq in The Cancer Genome Atlas Cohort (TCGA). TCGA data was obtained from the www.cbioportal.org platform [28,29]; Uterine Corpus Endometrial Carcinoma (TCGA, Nature 2013) [2]. NS: non-significant (p>0.05); *, **, ***, ****: *p*-values, respectively: <0.05; <0.01; <0.001; <0.0001. Dots depicted as crosses refer to tumors considered as NRF2 activated in the Cochin cohort, as shown on **Fig 3**. A. NQO1 expression and histological type (CCH). Type I and type II refer to histological subtypes (**Table 1**). B. NQO1 expression and TP53 staining. Altered TP53 staining considered both staining with overexpression or staining with loss of expression. C. NQO1 expression and molecular group (CCH). POLE: *POLE* exonuclease domain mutated tumors. MSI: *microsatellite instable-like* tumors. TP53: *TP53/copy-number high-*like tumors. CNL: *copy number low*-like tumors. Paired comparisons computed using post-hoc test adjusted for the risk α for multiple comparisons. POLE tumors were not included in statistical test because of the number of samples in the group (<5). D. NQO1 expression and molecular group (TCGA). POLE: *POLE* tumors. MSI: microsatellite instable tumors. Anova: analysis of variance. *p-*values are depicted in * to **** for graphical purposes. Paired comparisons computed using post-hoc test adjusted for the risk α for multiple comparisons. E. NQO1 expression and histological type within copy-number high group. TCGA data. *p*-value based on Student t-test. F. Event free survival and NQO1 expression. Kaplan-Meier curves. *p*-value based on two-sided log-rank test.

**Fig 5:**
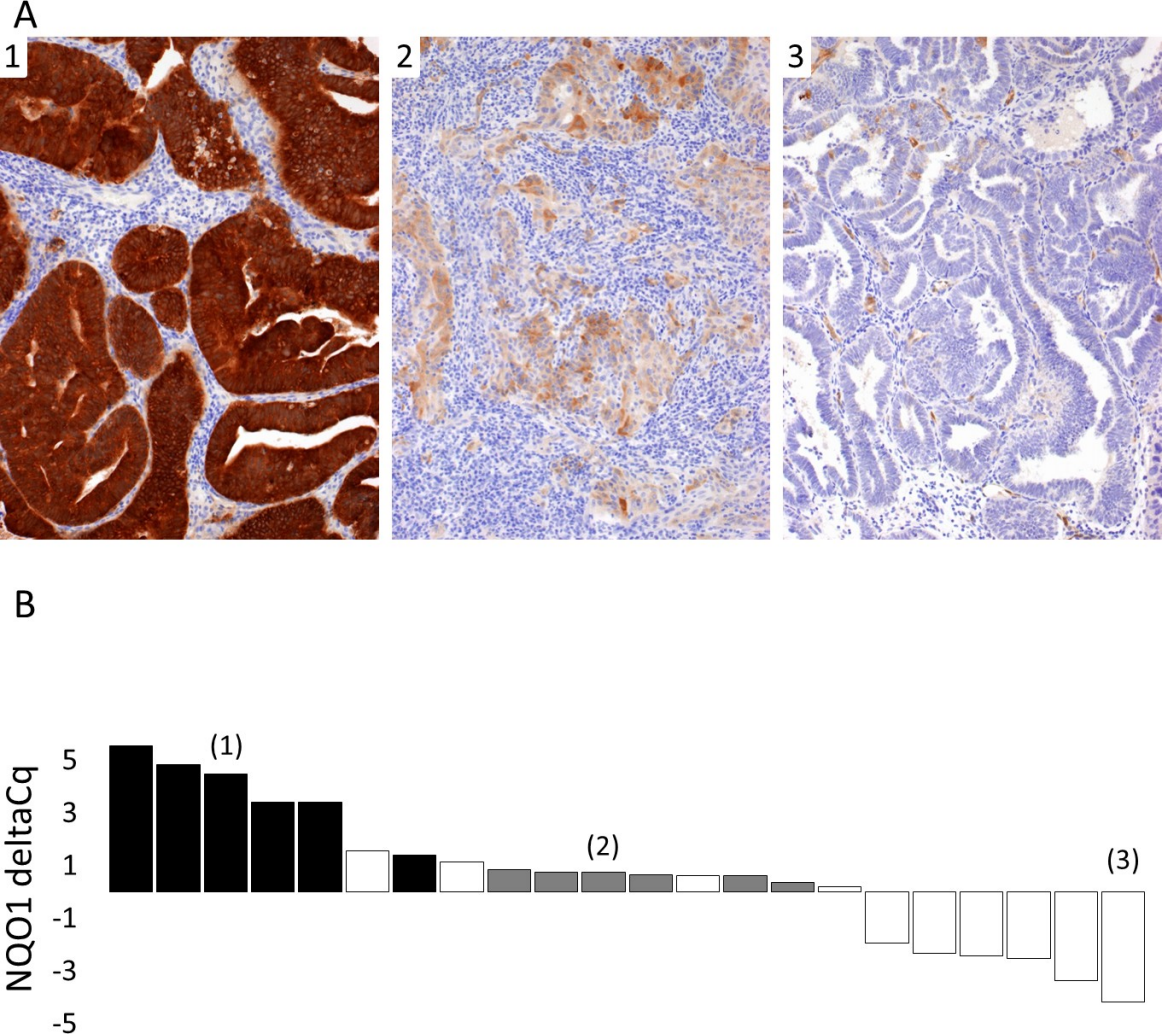
Correlation between NQO1 protein expression and NQO1 mRNA expression. A. Representative examples of NQO1 immunohistochemical assay. Primary NQO1 antibody was used (SIGMA-ALDRICH, HPA007308), revealed with Leica Bond Polymer Refine Detection System Kit. 10X objective. Sample (1): NQO1 high case. Sample (2): NQO1 intermediate case. Sample (3): NQO1 low case. B. NQO1 mRNA levels and NQO1 immunohistochemical assay results. NQO1 expression measured by quantitative RT-PCR is shown as barplot. *y-*axis display delta Cq expression. Black bars highlight samples with high NQO1 protein expression. Grey bars highlight samples with intermediate NQO1 protein expression. White bars highlight samples with low NQO1 protein expression. (1), (2), and (3) refer to samples for which NQO1 immunohistochemical assay results are provided on Fig 5A.

A subset of outlier tumors showed a low *NQO1* mRNA level, depicted in dark grey bars in **Fig 3** on the basis of a gap in the *NQO1* expression distribution. These tumors had variable mRNA levels of *GCLC* and *AKR1C3* and various NRF2 staining intensity (high: *N =* 2; intermediate: *N =* 4; low: *N =* 3). This prompted us to search for histological, pathological or molecular features associated with a low *NQO1* mRNA level. We observed a lower *NQO1* mRNA level in type II carcinoma (**Fig 4A**) and in tumors bearing a TP53 alteration in immunohistochemistry assay (**Fig 4B**). As shown on **Fig 4C**, we also observed a low *NQO1* mRNA level in the *TP53/CNH-like* molecular subgroup. This observation was consistent with RNA-sequencing data analysis from TCGA endometrial carcinoma dataset (**Fig 4D**) (data set from the www.cbioportal.org platform [28,29]; Uterine Corpus Endometrial Carcinoma (TCGA, Nature 2013) [2]). *GCLC* and *AKR1C3* mRNA levels were not decreased in the CNH molecular group (**S6 Fig**). Finally, because of the histopathological heterogeneity of the *TP53/CNH-like* molecular group, which includes type II carcinomas (*N* = 13, 56.5%), and also type I endometrial carcinomas (*N* = 9, 40.1%), we wondered whether a low *NQO1* mRNA level could be related to the histological type or estrogen receptor positivity. We did not observe any significant difference in our cohort (**S7 Fig**), but the statistical power was weak (*N =* 22). However, when exploring the TCGA CNH group, we observed a significantly lower *NQO1* mRNA level in serous carcinoma, compared to endometrioid carcinoma (**Fig 4E**). In order to confirm that a low NQO1 expression was indeed observed in *TP53/CNH-like* tumors despite the trend for higher NRF2 staining, we assessed NQO1 protein expression in 22 samples (**Fig 5**). The 5 samples with NQO1 mRNA level in the highest quartile were scored NQO1-high and 4 out of these harbored known NRF2 activating mutations. The 6 samples with NQO1 mRNA level in the lowest quartile were scored NQO1 weak/negative and belonged to the *TP53/CNH-like* molecular group. Intermediate levels of NQO1 mRNA expression (Q2, Q3) showed high (n = 1), intermediate (n = 6) or weak/negative (n = 4) NQO1 staining. These findings indicate a low mRNA level of *NQO1* in the *TP53/CNH-like* group and suggest that the mechanisms regulating *NQO1* mRNA level may vary within the *CNH* group depending on histological type.

Several studies reported the relations between NRF2 activation and PI3K pathway activation in endometrial carcinoma [13], or *KRAS* mutations in other tumor types[15,30]. In our cohort, no significant association was observed between *PIK3CA*, *PIK3R1*, or *PTEN* mutations, and *NQO1*, *GCLC*, or *AKR1C3* expression (**S8 Fig**). The only significant association found was a higher *NQO1* expression in *KRAS* mutated tumors (*p =* 0.033, **S9A Fig**). This association was probably due to a bias related to the high frequency of *KRAS* mutation in non-TP53 mutated tumors and was not confirmed when performing this analysis after removing *TP53/CNH*-like tumors (*p =* 0.26, **S9B Fig**). The analysis of the TCGA data further confirmed the absence of correlation between high NRF2 target expression and mutation of *KRAS*, *PIK3CA*, *PIK3R1* and *PTEN* (**S5 Fig**).

### Association between *NQO1* mRNA level and prognosis

Ninety patients were included in the survival analysis (EFS). Median follow-up was 24.1 months (interquartile range: [13.8;43.7]–reverse Kaplan-Meier method[31]). As shown in **Fig 3A**, nine samples had a low *NQO1* mRNA level. Patients belonging to this subgroup exhibited a poor prognosis (1-years EFS = 47.6%, 95% confidence interval (95%CI) [22.0; 100.0]–**Fig 4F**) when compared to patients without low *NQO1* mRNA level (1-years EFS = 84.0%, 95%CI [75.8; 93.2] (Univariate hazard ratio (HR) = 4.66, 95%CI[1.80; 12.1], *p =* 0.002)). However, in an adjusted Cox regression model that includes the *TP53/CNH-like* molecular group as main confounder, low *NQO1* mRNA level was not independently associated with poorer survival (adjusted-HR = 1.7, 95%CI[0.6; 5.3], *p =* 0.3), while *TP53/CNH-like* molecular subgroup was (HR = 4.02, 95%CI[1.44; 11.3], *p =* 0.008). These results show an association between low *NQO1* mRNA level and poor outcomes but were underpowered to explore subgroup analysis according to *NQO1* mRNA level within the *TP53/CNH-like* molecular group.

## Discussion

We report here a comprehensive analysis of the NRF2 activation process in endometrial carcinoma using genes expression data, genetic characterization, protein expression/subcellular location assay. In contrast with previous reports [16,32], despite 24.7% of samples showing a high NRF2 nuclear staining, we observed a cluster of only 3 out of 90 (3%) cases with NRF2 activating mutations and a concomitant overexpression of three core target genes. This finding suggests that NRF2 activation driven by molecular alterations affecting NRF2/KEAP1 binding is a rare event in endometrial carcinoma. On the other hand, we observed a strikingly low NQO1 expression in aggressive *TP53/CNH-like* tumors. This finding was also in contrast with conventional models suggesting that the high ROS production in aggressive tumor cells activates NRF2 in order to increase detoxification genes expression, such as NQO1, and to sustain a high proliferative rate [4].

We have observed a strong trend toward more NRF2 highly stained tumors within the *TP53/CNH-like* group, known to be of poor prognosis [2], consistent with previous reports describing higher NRF2 expression with advanced stage and high tumor grade in endometrial carcinomas [32]. However, we did not observe any association between high NRF2 staining and its target genes expression. This could be due to several issues. First, the NRF2 antibodies specificity has been described to be potentially limited [17]. No *gold-standard* is for now available for NRF2 immunohistochemistry assay. In some reports, NRF2 staining has been observed in the cytoplasm. We considered the staining into the nuclei as consistent with NRF2 physiology, NRF2 being described as constantly degraded in the cytoplasm [3]. The observation of cytoplasmic staining could be related to non-specific staining related to antibody specificity issues [17] or different antigen retrieval methods. On the other hand, the sequencing results were consistent with TCGA *in-silico* data analysis, based on exome sequencing, while the qPCR expression results led to similar conclusions when faced to TCGA RNA-sequencing data. Our results indicate that assessing NRF2 core target genes expression appears more reliable than immunohistochemistry to assess NRF2 activation.

Recent studies have reported the involvement of NRF2 in biological processes others than its canonical role in cell detoxification and metabolism reprogramming. As reported by Kalo *et al*. [33] and more recently by Walerych *et al*. [34], in tumor cells affected by *TP53* missense mutations, NRF2 cooperates with *TP53* mutant isoforms and colocalizes on proteasome gene promoters, subsequently inhibiting multiple tumor suppressive pathways and driving an aggressive phenotype. Importantly, this phenomenon precludes NRF2 binding on ARE promoters, and induces a downregulation of various AOS genes, more particularly phase II detoxification genes, such as *NQO1* or *HMOX1*, or *SLC7A11* [34,35]. Subsequently, these tumors were shown to reach higher ROS levels. The observation that endometrial *TP53/CNH-like* tumors, especially tumors with serous histology, have very low NQO1 mRNA levels could be related to NRF2/TP53 cooperation and may have interesting clinical implications. The observation that serous carcinoma are initially highly sensitive to chemotherapy [36] could be related to this NRF2 downregulation, which would limit the detoxification capacities of the tumor cells. NRF2/TP53 cooperation were shown to be targeted *in vivo* by specific combination therapies [34,35]. For instance, the addition of a missense mutant TP53 inhibitor APR-246 to the proteasome inhibitor Carfilzomib has been demonstrated to be active *in vivo* on triple-negative breast cancer models, a tumor carrying a molecular background similar to CNH endometrial carcinoma [34]. Another example is the sensitivity of these tumors to inhibitors of the cystine/glutamate antiporter [35], which deplete GSH in TP53 mutated cells, resulting in an oxidative burst and cell death. Indeed, the identification of such biological oncogenic process in endometrial tumors could help to select patients with particularly poor outcomes who could take advantage of these new therapeutic approaches. On the other hand, *NQO1* regulation may involve other transcriptional factors and its low expression in a subset of endometrial carcinomas might be related to biological processes independent of NRF2: an extensive chromatin and transcriptomic analysis of these tumors would be required to determine whether NRF2 transcriptional program is indeed skewed in TP53 mutated serous tumors. Finally, because the interplay described between NF-kB and NRF2 [37], tumors with low NQO1 expression belonging to the *TP53/CNH-like* group could be affected by higher NF-kB activity, which could imply more inflammation. Further studies are warranted to confirm whether this feature could participate to aggressiveness of this molecular subgroup.

In conclusion, our work suggests that *NFE2L2/KEAP1* mutations leading to a strong NRF2 activation is a rare event in endometrial carcinoma, and that a subset of *TP53/CNH-like* endometrial carcinoma with serous histology have low *NQO1* expression, that could be related to a NRF2/TP53 cooperation. This last phenomenon could drive their aggressiveness, although making them initially more sensitive to chemotherapy. If confirmed, new therapeutic approaches and combinations should be investigated in this subgroup of CNH endometrial carcinoma.

## Supporting information

S1 Fig Study flow chart. NGS: next generation sequencing, performed on IonTorrent PGM device. FFPE: formalin-fixed paraffin embedded. cDNA: coding DNA after reverse transcription. *one patient with 2 samples processed for sequencing, without qualitative changes on results. Sample with highest cellularity and best conservation was processed for RNA extraction.
(PNG)

S2 Fig Distribution of NRF2 target genes expressions. NQO1, GCLC and AKRC3 expressions measured by quantitative RT-PCR are shown as 3D scatter plot. Axis: Color palette from dark blue to dark red follow NQO1 deltaCq expression. Plan is the NQO1 bidimensionnal regression plan according to GCLC and AKR1C3 expression (Pearson correlation coefficient for NQO1/AKR1C3 = 0.41, NQO1/GCLC = 0.41 and GCLC/AKR1C3 = 0.55).
(PNG)

S3 Fig Endometrial carcinoma clustering based on NRF2 target genes expressions, based on principal component analysis. Clustering was based on principal component analysis. PC1 and PC2: principal component 1 and 2. CNL: *copy-number low*-like tumors. MSI: *microsatellite instable-like* tumors. NRF2: tumors with NRF2 activating mutations (**Fig 3** and **Table 2**), assumed to be clonal. NRF2_SC: tumors with NRF2 activating mutations (**Fig 3** and **Table 2**), assumed to be sub-clonal on the basis of low allele ratio. POLE: *POLE* exonuclease domain mutated tumors. TP53: *TP53/copy-number high*-like tumors.
(PNG)

S4 Fig Endometrial carcinoma clustering based on NRF2 target gene expression in the TCGA cohort, based on principal component analysis. Clustering was based on principal component analysis. PC1 and PC2: principal component 1 and 2. POLE: POLE molecular tumor group; MSI: microsatellite instable tumor; NRF2: tumors bearing a missense mutation within the NRF2/KEAP1 binding domains on NFE2L2 (DLG and ETGE motifs) or missense mutation on *KEAP1* (aa 324–597) coding sequences or *KEAP1* truncating mutations. Principal component analysis used RNA-seq RSEM (V2) data available at the www.cbioportal.org portal.
(PNG)

S5 Fig Endometrial carcinoma clustering based on NRF2 target gene expression in the TCGA cohort—heatmap. Unsupervised hierarchical clustering depicted using heatmap. Data: RNA-seq RSEM (V2) gene expression data available at the www.cbioportal.org portal. Genes considered belong to a NRF2 transcriptional signature including genes overlapping between two gene lists: genes repressed by a NFE2L2 siRNA-based silencing in A549 cells, a lung cancer cell line with a KEAP1 mutation (Mitsuishi et al. Cancer Cell 2012 Jul. 10;22(1):66–79) & genes significantly overexpressed in lung carcinoma *KEAP1*- or *NFE2L2*- mutated (TCGA dataset, Nature 2014–230 cases) versus double wild-type carcinoma. Annotations depict mutations in signaling pathway of specific interest.
(PNG)

S6 Fig NRF2 target gene expressions according to endometrial carcinoma molecular group in Cochin Hospital Cohort and in The Cancer Genome Atlas Cohort–full results. **A-C**: Cochin Hospital cohort (CCH). POLE: *POLE* exonuclease domain mutated tumors. MSI: *microsatellite instable-like* tumors. TP53: *TP53/copy-number high-*like tumors. CNL: *copy number low*-like tumors. Note that the 3 outliers in NQO1 and AKR1C3 plots are samples bearing clonal NRF2 pathway alterations (**Fig 3**). *p*-values are estimated using a post-hoc test adjusted for the risk α for multiple comparisons. Note that POLE tumors were not included in the post-hoc test because of the number of samples in the group (<5). **D-F**: The Cancer Genome Atlas Cohort (TCGA). POLE: *POLE* tumors. MSI: microsatellite instable tumors. NS: p > 0.05 *: p ≤ 0.05. **: p ≤ 0.01. ***: p ≤ 0.001. ****: p ≤ 0.0001. ANOVA: analysis of variance. *p*-values depicted in * to **** for graphical purposes and estimated using post-hoc test adjusted for the risk α for multiple comparisons.
(PNG)

S7 Fig NQO1 expression according to estrogen receptor positivity and histological type in *TP53/CNH-like* tumors in Cochin Hospital cohort. Type I and type II carcinoma: see Table 1 for distribution and details. Estrogen receptor positivity: assessed using standard immunohistochemistry assay: tumors were considered positive if staining intensity was ≥+ in more than 10% tumors cells.
(PNG)

S8 Fig NRF2 core target genes expressions according to KRAS and PI3K pathways mutations in Cochin Hospital cohort. CCH: Cochin Hospital cohort.
(PNG)

S9 Fig NQO1 expression according to KRAS mutation in non-*TP53/CNH-like* tumors in Cochin Hospital cohort. (PNG)

S1 Method Targeted sequencing panel and coverage analysis. (XLSX)

S2 Method NRF2 targeted sequencing panel and coverage analysis. (XLSX)

S3 Method Variant Caller parameters. (XLSX)

S1 Dataset Supporting information file. Clinical, histological and molecular characteristics of the 90 EC included in the study.
(XLSX)
