# Supplementary figures and images for: The NRF2 transcriptional target NQO1 has low mRNA levels in TP53-mutated endometrial carcinomas
Source: PLoS One. 2019 Mar 25;14(3):e0214416. doi: 10.1371/journal.pone.0214416 (PMC6433262; doi:10.1371/journal.pone.0214416)

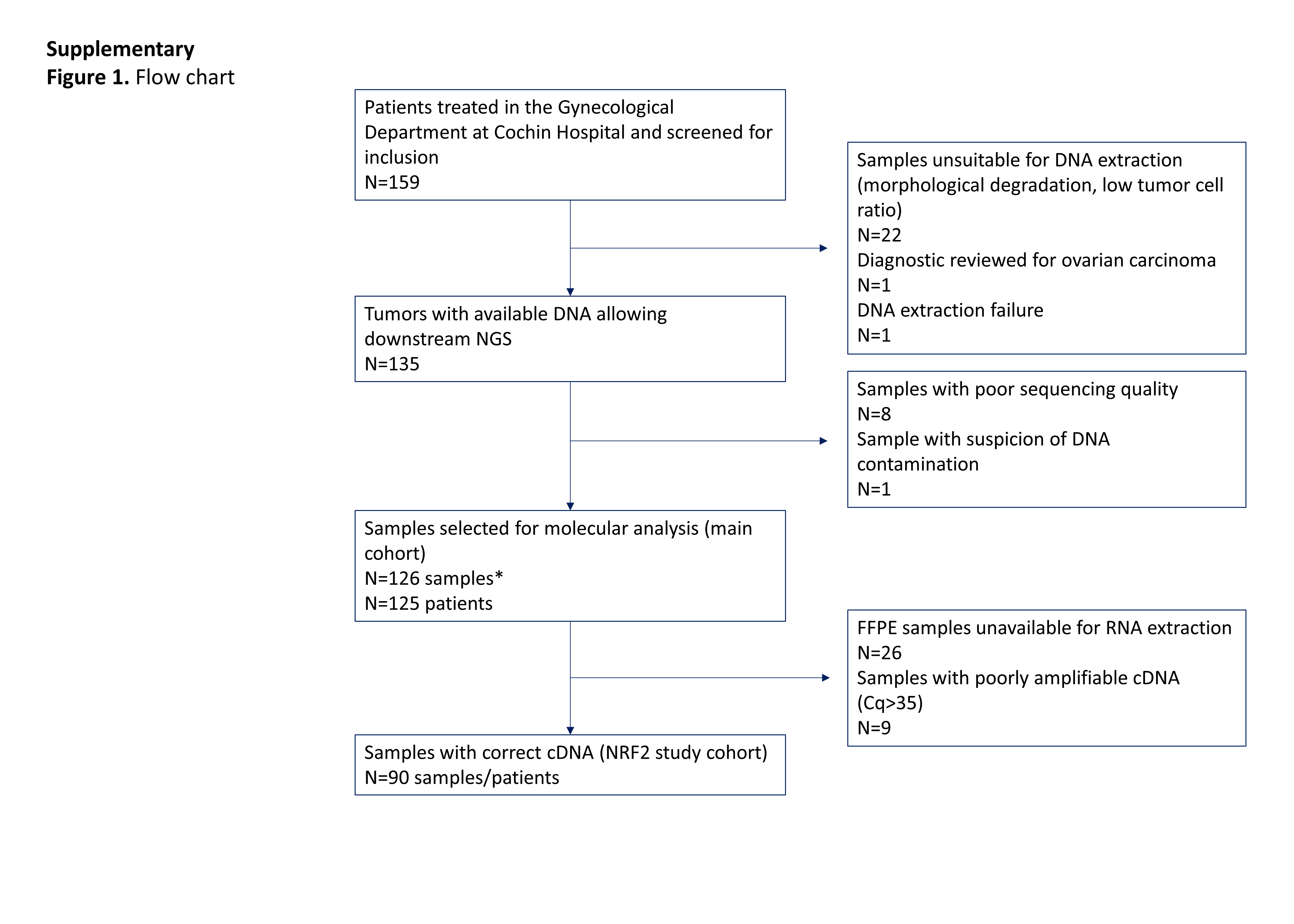

Supplement: S1 Fig — NGS: next generation sequencing, performed on IonTorrent PGM device. FFPE: formalin-fixed paraffin embedded. cDNA: coding DNA after reverse transcription. *one patient with 2 samples processed for sequencing, without qualitative changes on results. Sample with highest cellularity and best conservation was processed for RNA extraction. (PNG) [file pone.0214416.s001.png]

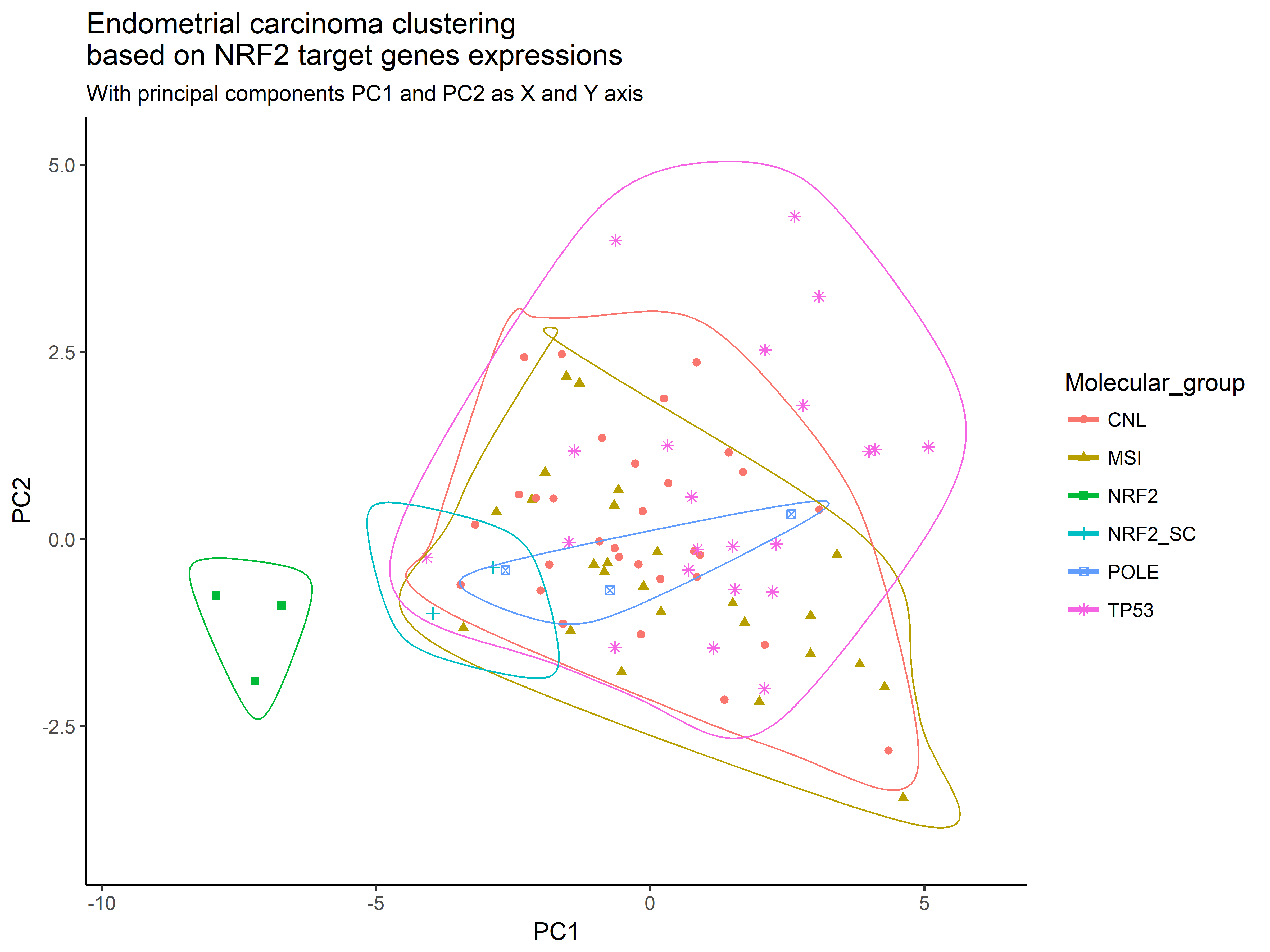

Supplement: S3 Fig — Clustering was based on principal component analysis. PC1 and PC2: principal component 1 and 2. CNL: copy-number low-like tumors. MSI: microsatellite instable-like tumors. NRF2: tumors with NRF2 activating mutations (Fig 3 and Table 2), assumed to be clonal. NRF2_SC: tumors with NRF2 activating mutations (Fig 3 and Table 2), assumed to be sub-clonal on the basis of low allele ratio. POLE: POLE exonuclease domain mutated tumors. TP53: TP53/copy-number high-like tumors. (PNG) [file pone.0214416.s003.png]

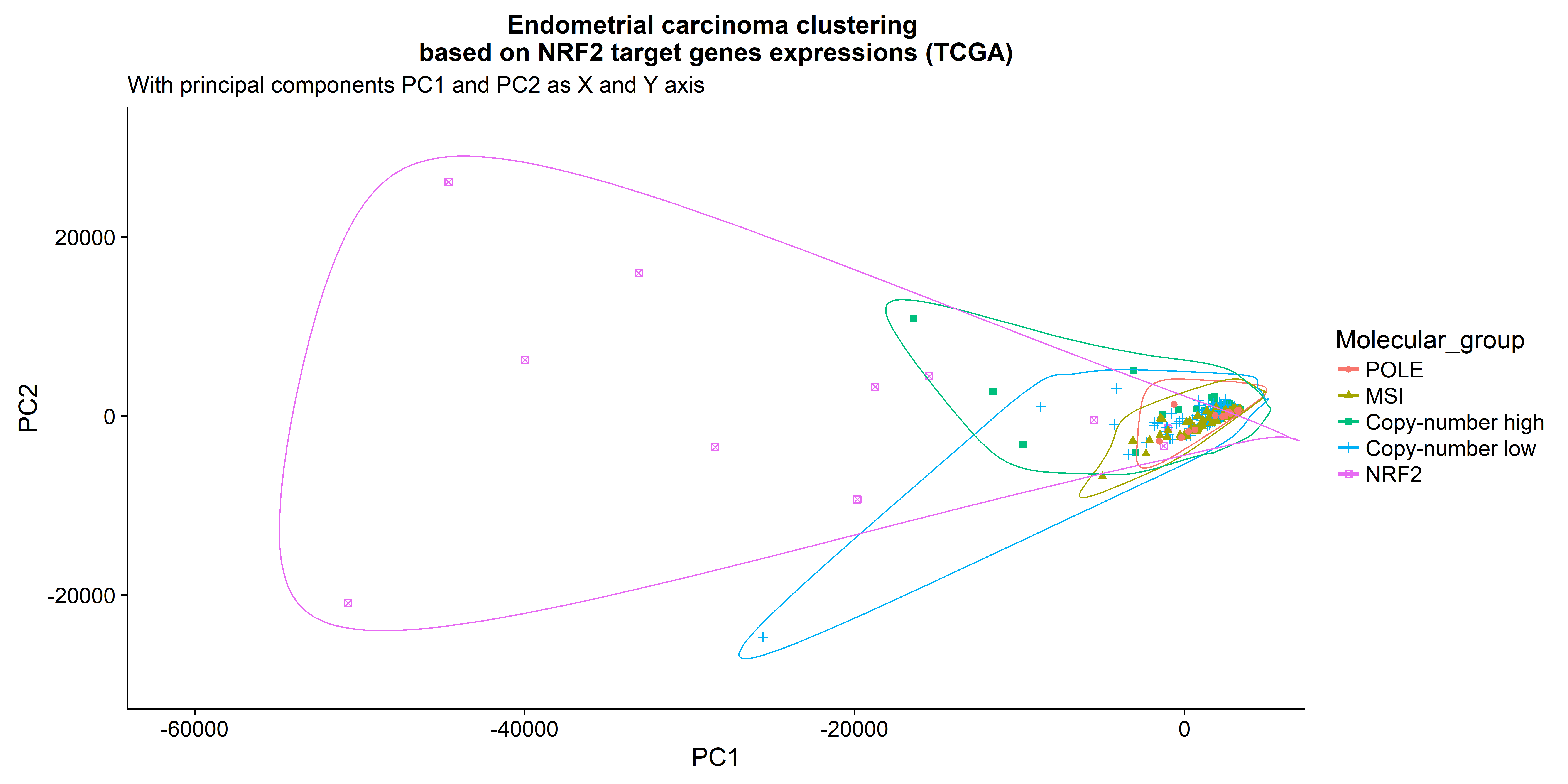

Supplement: S4 Fig — Clustering was based on principal component analysis. PC1 and PC2: principal component 1 and 2. POLE: POLE molecular tumor group; MSI: microsatellite instable tumor; NRF2: tumors bearing a missense mutation within the NRF2/KEAP1 binding domains on NFE2L2 (DLG and ETGE motifs) or missense mutation on KEAP1 (aa 324–597) coding sequences or KEAP1 truncating mutations. Principal component analysis used RNA-seq RSEM (V2) data available at the www.cbioportal.org portal. (PNG) [file pone.0214416.s004.png]

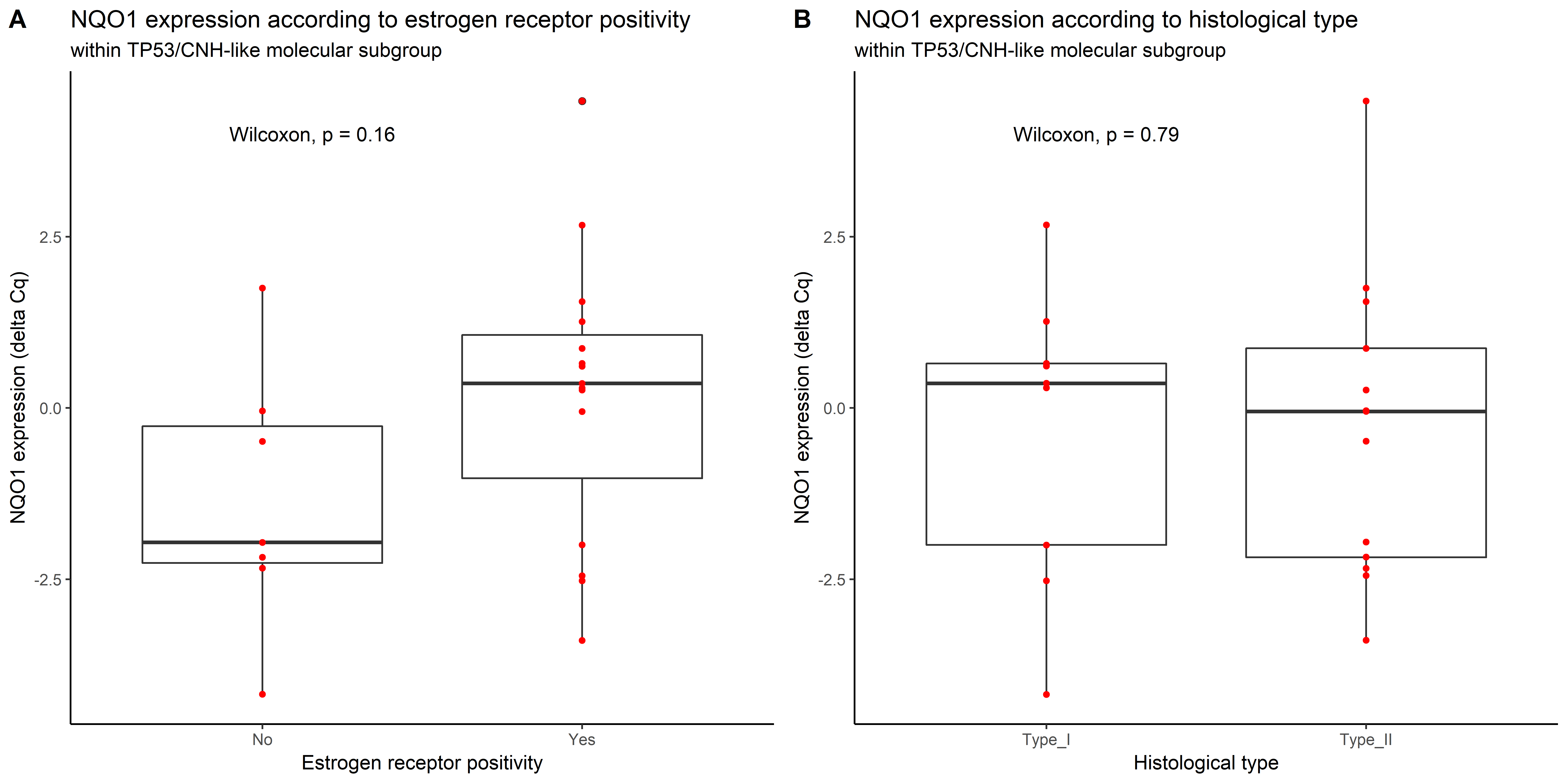

Supplement: S7 Fig — Type I and type II carcinoma: see Table 1 for distribution and details. Estrogen receptor positivity: assessed using standard immunohistochemistry assay: tumors were considered positive if staining intensity was ≥+ in more than 10% tumors cells. (PNG) [file pone.0214416.s007.png]

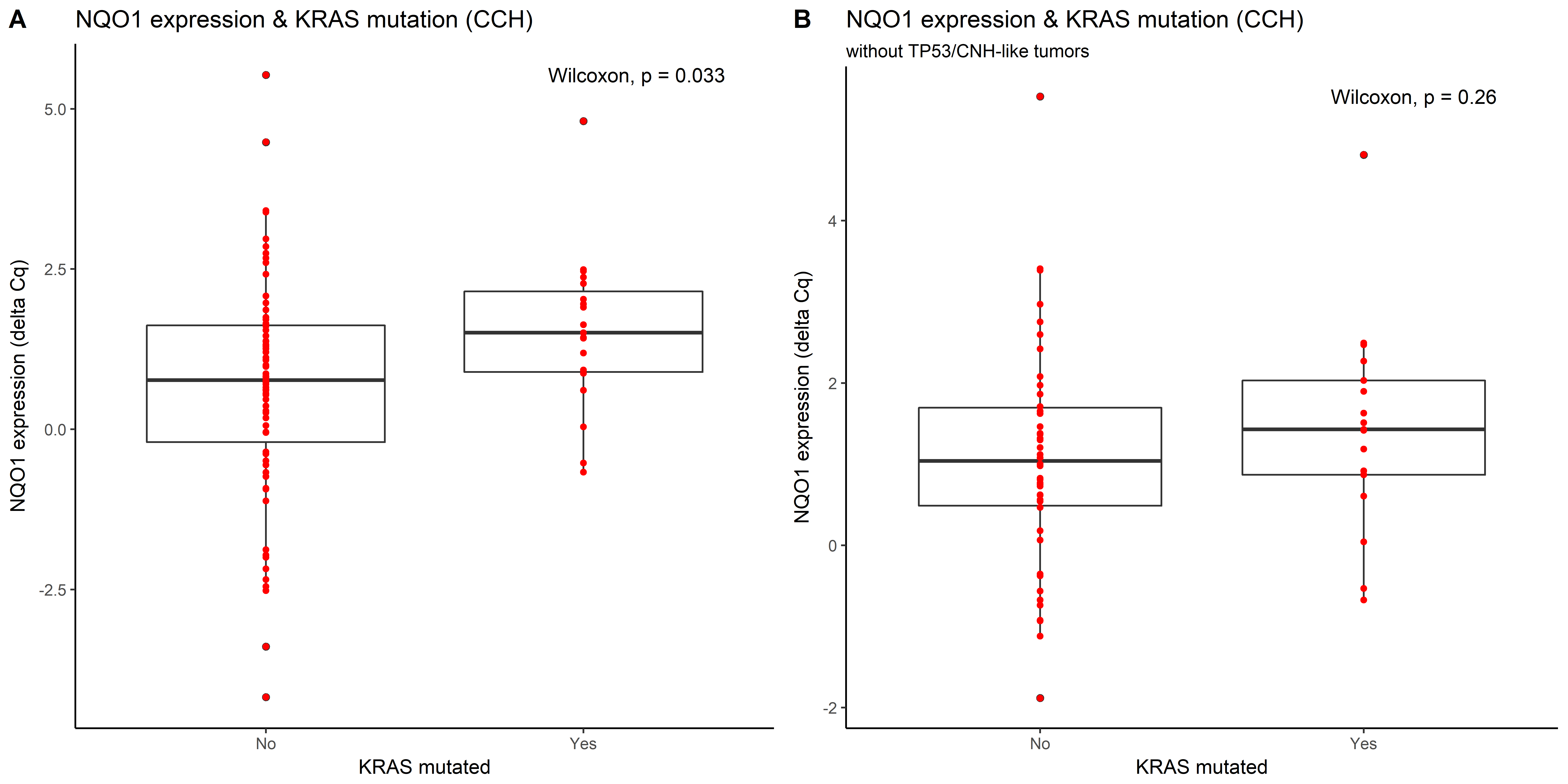

Supplement: S9 Fig — (PNG) [file pone.0214416.s009.png]
